# Self-reported health and socio-economic inequalities in England, 1996–2009: Repeated national cross-sectional study

**DOI:** 10.1016/j.socscimed.2015.05.026

**Published:** 2015-07

**Authors:** Hendramoorthy Maheswaran, Emil Kupek, Stavros Petrou

**Affiliations:** a Division of Health Sciences, University of Warwick Medical School, Coventry, UK; b Malawi-Liverpool-Wellcome Trust Clinical Research Programme, Blantyre, Malawi; c Department of Public Health, Federal University of Santa Catarina, Florianopolis, Brazil

**Keywords:** England, EQ-5D utility scores, GHQ, Self-assessed health, Social class, Social inequities

## Abstract

Tackling social inequalities in health has been a priority for recent UK governments. We used repeated national cross-sectional data for 155,311 participants (aged ≥16 years) in the Health Survey of England to examine trends in socio-economic inequalities in self-reported health over a recent period of sustained policy focus by successive UK governments aimed at tackling social inequalities in health. Socio-economic related inequalities in self-reported health were estimated using the Registrar General's occupational classification (1996–2009), and for sensitivity analyses, the National Statistics Socio-Economic Classification (NS-SEC; 2001–2011). Multi-level regression was used to evaluate time trends in General Health Questionnaire (GHQ-12) scores and bad or very bad self-assessed health (SAH), as well as EQ-5D utility scores.

The study found that the probability of reporting GHQ-12 scores ≥4 and ≥ 1 was higher in those from lower social classes, and decreased for all social classes between 1997 and 2009. For SAH, the probability of reporting bad or very bad health remained relatively constant for social class I (professional) [0.028 (95%CI: 0.026, 0.029) in 1996 compared to 0.028 (95%CI: 0.024, 0.032) in 2009], but increased in lower social classes, with the greatest increase observed amongst those in social class V (unskilled manual) [0.089 (95%CI: 0.085, 0.093) in 1996 compared to 0.155 (95%CI: 0.141, 0.168) in 2009]. EQ-5D utility scores were lower for those in lower social classes, but remained comparable across survey years. In sensitivity analyses using the NS-SEC, health outcomes improved from 2001 to 2011, with no evidence of widening socio-economic inequalities. Our findings suggest that socio-economic inequalities have persisted, with evidence of widening for some adverse self-reported health outcomes.

## Highlights

• Study examines trends in social inequalities in health in England (1996–2009).
• Study uses a range of self-reported measures of health status.
• Study examines net changes using national data from 155,311 participants.
• Findings suggest potential widening of socio-economic inequalities in health.

## 1 Introduction

A plethora of research highlights that those of lower socioeconomic position are at increased risk of adverse health outcomes, including cardiovascular disease (Mackenbach et al., 2003), cancers (Coleman et al., 2004; Forrest et al., 2013; Parikh et al., 2003), mental health problems (Jokela et al., 2013), and unhealthy lifestyle behavioural factors (Devaux and Sassi, 2013; Rumble and Pevalin, 2013). This is likely to require increased expenditure the by health services and result in reduced productivity (Marmot et al., 2010; WHO, 2008). It is also generally accepted that greater availability of economic resources will tend to result in improvements in population health (Hurd and Kapteyn, 2003). However, it is increasingly felt that unequal distributions of resources within populations drive social inequalities in health (Babones, 2008; Wilkinson and Pickett, 2006). Evidence from the UK, and other European countries, suggests a widening socioeconomic gradient in adverse health outcomes during the latter 20th century (Black et al., 1980; Mackenbach et al., 2003; Marmot et al., 1991). However, it was not until the late 1990's, after the publication of the Acheson Report (Acheson, 1998), that tackling social inequalities in health became a priority for health policy in the UK.

The period of the most recent UK Labour governments (1997–2010) saw increased focus and investment in preventing poor health through addressing underlying determinants of health inequalities, including educational attainment (OECD, 2009), unemployment, housing and through targeting deprived communities (DoH, 2003), supported by one of the longest time periods of sustained economic prosperity (Benati, 2008) that generated additional spending for the public sector and increased real incomes across the income spectrum (Browne and Phillips, 2010). There are concerns these efforts and investments did not have their desired effects (Mackenbach, 2011). Whilst there is some evidence suggesting the UK National Health Service (NHS) provided a more equitable service as a result (Cooper et al., 2009), this has not translated into a reduction in social inequalities in health (Devaux and Sassi, 2013; Jokela et al., 2013; Rumble and Pevalin, 2013; Scholes et al., 2012; Thomas et al., 2010).

The impact of economic downturns on social inequalities in health has been widely studied, with conflicting findings. In Japan, economic stagnation during the late 1990's and early 2000's was associated with a narrowing in social inequalities in health (Kachi et al., 2013), whilst in New Zealand, South Korea and Russia periods of economic hardships were associated with widening in social inequalities in health (Blakely et al., 2008; Hong et al., 2011; Plavinski et al., 2003), with evidence from European countries suggesting minimal changes during economic downturns (Kunst et al., 2005). The range of findings reflects differences in government spending, welfare state provision and changes in income during these periods of economic downturns, and the complex interactions between these factors (Benzeval and Judge, 2001; Eikemo et al., 2008; Wilkinson and Pickett, 2006). What has been less studied are trends in social inequalities in health across economic cycles, and when studied, has predominantly focussed on objective measures of health outcome, including mortality and life expectancy, risk factors for poor health and use of health services (Devaux and Sassi, 2013; Jokela et al., 2013; Rumble and Pevalin, 2013; Scholes et al., 2012; Thomas et al., 2010).

Measuring the population's health can be achieved in a number of ways, including monitoring changes in objective measures of health outcomes and health services use, metrics used by the authorities in the UK to monitor trends in social inequalities in health (DoH, 2003). However, at the population-level policy concerns should not be limited to objective measures of health, but encompass wider impacts on health, including health-related quality of life and mental well-being (WHO, 1986). However, measuring the health of the population is complex, and choices have to be made on whom we choose to sample to represent the population, and the tools used to measure and quantify the population's health status. The use of self-reported measures of health status in national health surveys offer an alternative approach to monitoring trends and inequalities in health over time, and investigating the impact of major policy initiatives (Fitzpatrick et al., 1992; Layard, 2010). In addition, the impact of national policy initiatives may not immediately translate into improvements in mortality or objective measures of morbidity, whilst self-reported measures of health provide reliable predictors of the health of the population (Benjamins et al., 2004; Jylha, 2009), and capture outcomes of relevance to individuals (Patrick and Chiang, 2000).

A range of self-reported measures of health have been developed. However, each differs in what it measures, the error in its measurement, its ability to differentiate health status of different population sub-groups, and its potential for capturing biases amongst responders and non-responders (Etches et al., 2006; Macran et al., 2003; Ziebarth, 2010). In addition, these tools use different approaches to measuring health status of individuals, and in the subsequent quantification of good and poor health (Goldberg et al., 1998; Manor et al., 2000). The objective of this study was to use a range of self-reported measures of health status to estimate secular trends in socio-economic related health inequalities in England over a recent 14 year period of sustained policy focus by UK governments on health inequalities. The study analyses national data for representative samples of the English population and uses a range of self-reported health status measurement tools, thereby maximising the relevance of the findings to the broader debates about socio-economic inequalities in health.

## 2 Methods

### 2.1 Data source

Data for this study came from the Health Survey for England (HSE), a series of annual surveys of nationally representative non-institutionalised residents in England. A detailed description of methods applied by the HSE is provided elsewhere (Mindell et al., 2012). The survey consists of a core set of demographic, economic and health questions asked each year. In addition, different annual surveys focus on a single or multiple health problems, and/or boost samples that allow investigation of specific population subgroups that would otherwise be under-sampled. The HSE adopts a two-stage stratified random sampling process using a Postcode Address File as the primary sampling unit (PSU). Individuals selected in one year are excluded for the following 3 years, although the relatively small proportions recruited from the eligible population makes it unlikely individuals would be recruited in subsequent years (Mindell et al., 2012). Adult interview response rates have fallen since introduction from approximately 70% in surveys undertaken in the 1990's to a plateau of approximately 60% in the late 2000's (Mindell et al., 2012). This analysis is based on the annual surveys undertaken from 1996 to 2011 and excludes additional participants sampled for the purpose of boosting population subgroups (boost samples of adults aged ≥65 years in 2000 and 2005 and black and minority ethnic groups in 1999 and 2004). This study uses secondary data with no participant identifiers, and therefore ethical approval was not required.

### 2.2 Measures of health status

#### 2.2.1 General Health Questionnaire

The General Health Questionnaire (GHQ) was developed in the 1970's and provides a measure of current mental health (D. Goldberg and Williams, 2000). There are four versions of the GHQ, with the shorter 12-item version (GHQ-12) used in the HSE. The GHQ-12 has been widely used in many national surveys for measuring psychological well-being, and has been found to be negatively correlated with global measures of health-related quality of life (HRQoL) (D. P. Goldberg et al., 1997). Responses to the 12 items are answered on a four-category Likert scale with response categories of ‘not at all’, ‘no more than usual’, ‘rather more than usual’ and ‘much more than usual’; the first and second categories are given a score of 0, and the third and fourth categories a score of 1. Scores from the 12 items are summed to generate a total score out of 12. Responses to the GHQ-12 were dichotomised for the purposes of this study. However, there are no strict guidelines on the thresholds for dichotomisation. Previous studies have suggested that the mean GHQ-12 score for the study population can be used as a threshold (Goldberg et al., 1998), whilst GHQ-12 scores ≥4 have been found to indicate poor mental health (D. Goldberg and Williams, 2000). In the HSE study years where GHQ-12 data were collected, the mean score was approximately 1.3. For this study, two thresholds were applied: (1) GHQ-12 score ≥4; (2) GHQ-12 score ≥1. GHQ responses were collected from participants every study year with the exception of 1996, 2007 and 2011. This analysis accounts for errors in the GHQ data (UK Data Archive, 2011).

#### 2.2.2 Self-assessed health (SAH)

Self-assessed health is a commonly used measure of health status that asks individuals to rate their general health on a four or five-point Likert scale. The measure provides a valid and reliable assessment of overall health status, and has been found to be predictive of future health outcomes when used in national population health surveys (Idler and Benyamini, 1997; Miilunpalo et al., 1997). The HSE uses the five-point Likert scale, with responses coded as: very good; good; fair; bad; or very bad. External evidence suggests that responders' health status gradually worsens as responses to this measure move from “very good” to “very poor” (Manderbacka et al., 1998). Responses were dichotomised using two different approaches to classifying self-assessed health; firstly, very good, good or fair versus bad or very bad (Mantzavinis et al., 2005); and secondly, very good or good versus fair, bad or very bad. The HSE collected responses to SAH from participants every study year.

#### 2.2.3 EQ-5D

The EQ-5D is a generic HRQoL instrument, consisting of two principal measurement components, a descriptive system and a visual analogue scale (EuroQol, 1990). This study concentrates on the EQ-5D descriptive system as the visual analogue scale, a scale (similar to a thermometer) ranging from 100 (best imaginable health state) to 0 (worst imaginable health state), is not routinely included in the HSE. The EQ-5D descriptive system defines the subject's HRQoL on the day of completion in terms of five dimensions: ‘mobility’, ‘self-care’, ‘usual activities’, ‘pain/discomfort’ and ‘anxiety/depression’, each of which can take one of three responses (no problems; some or moderate problems; or severe or extreme problems). In the study years where the EQ-5D was used (1996, 2003–2006, 2008, 2010, and 2011), study subjects were always asked to complete the EQ-5D descriptive system. Responses to this descriptive system can theoretically generate 243 (3^5^) different health states. For purposes of this investigation, a UK specific tariff [York A1] was applied to each set of responses to the descriptive system in order to generate an EQ-5D utility score (a preference-weighted HRQoL outcome) for each participant. The York A1 tariff set was calculated from a time trade-off study of 3337 members of the UK general population (Dolan, 1997). It generates utility scores ranging between −0.594 and 1.0, with 1.0 corresponding to “perfect health” and 0 representing a health state considered to be equivalent to death.

### 2.3 Measures of socio-economic position

The HSE classifies social class using either the Registrar General's occupational classification (Szreter, 1984) or the National Statistics Socio-Economic Classification (NS-SEC) (Rose and Pevalin, 2003) or both. For the primary analyses, encompassing all study years until 2009, the social class of the head of household was classified according to the Registrar General's occupational classification, which contains the following categories: ‘I: Professional’; ‘II: Managerial/technical’; ‘IIINM: Skilled non-manual’; ‘IIIM: Skilled manual’; ‘IV: Semi-skilled manual’; ‘V: Unskilled manual’; or ‘Other’ (including armed forces). From 2001 onwards, the HSE also began recording the NS-SEC classification of the head of household, which contains the following categories: ‘managerial/professional’; ‘intermediate’; ‘small employers/own account workers’; ‘lower supervisory/technical’; ‘semi-routine’; and ‘other’. For both classifications, the HSE classifies retired individuals according to the last main occupation. Current evidence suggests the NS-SEC does not follow a hierarchical structure with regards to social class, and exhibits a weaker relation to health outcomes (Chandola, 2000). We therefore undertook sensitivity analyses investigating secular trends (2001–2011) for each of the health outcomes across NS-SEC categories.

### 2.4 Individual level covariates

As the purpose of the study was to investigate the trends in self-reported health status across socio-economic groups, we controlled for a range of individual-level covariates shown to have an independent association with the outcomes of interest (Maheswaran et al., 2013). These included sex, age marital status, educational attainment, income, smoking status, alcohol consumption and body mass index (BMI). The individual-level covariates were categorised using the approaches provided by the HSE developers, primarily to ensure comparability across the HSE years. For income, the annual household income was categorised into quintiles, after being deflated and equivalised using the McClements equivalence scale (McClements, 1977). Body mass index (BMI) was categorized as BMI <18.5 kg/m^2^; BMI 18.5–24.9 kg/m^2^ (reference category); BMI 25–29.9 kg/m^2^; BMI 30–34.9 kg/m^2^; and BMI 35–39.9 kg/m^2^. Cigarette smoking was categorized in terms of whether study participants were never ever smokers (reference category); light smokers, <10/day; moderate smokers, 10 to <20/day; heavy smokers, 20 + a day; ex-smokers; or didn't know their smoking status. Alcohol consumption was categorised into never ever drinkers (reference category); not at all in the last 12 months; once or twice a year; once every couple of months; once or twice a month; once or twice a week; three or four days a week; five or six days a week; almost every day; or ex-drinker.

### 2.5 Statistical analysis

Multi-level logistic and linear models were constructed for the binary (GHQ-12, self-assessed health) and continuous (EQ-5D utility score) outcomes, respectively. Variation between survey respondents within the same PSU was modelled as the first level, whereas variation across PSUs acted as the second level in all multivariate analyses. The level weights were adjusted to equal the inverse probability of selecting a particular PSU and the survey respondents within the PSU. Multi-level models with both fixed and random effects, survey weights, and multivariate adjustment for socio-demographic and behavioural health risk variables, were used to estimate time trends for the EQ-5D utility score for each social class. The statistical package GLLAMM was used for the binary outcome analyses with random effects and aforementioned multivariate adjustment, as well as to estimate the marginal distributions of posterior probabilities (Rabe-Hesketh and Skrondal, 2008). Time trend analysis for each social class used weighted regression of the marginal distributions with the weights equalling the inverse of the variance for the margin estimates. We incorporated survey weights, when provided (2003–2011), in all analyses to allow samples to be representative of the English population. A statistically significant change between calendar year estimates was defined by non-overlapping 95% confidence intervals of the predicted outcomes in fully adjusted regression models. For each regression, the predicted outcome was a conditional posterior expectation (mean for the EQ5D and log odds plotted as probabilities for binary outcomes) given the mean values of the covariates for each calendar year.

Three types of models were run: the first adjusted for age and sex; the second also adjusted for ethnicity, marital status, educational attainment, and income (equivalised for household composition); whilst the third additionally adjusted for smoking status, alcohol consumption and body mass index (BMI). The findings of the third sets of models are presented in the main text, with findings from the other sets of models presented in online appendices. All analyses were conducted using Stata MP version 13 (StataCorp LP, College Station, TX, 2013). For variables where there was missing data, missing values were coded as a separate category, which amounts to single imputation; multiple imputation was not undertaken because of the large samples used for analysis and consequently the high computational time.

## 3 Results

Table 1 presents descriptive statistics for the socio-demographic characteristics of all participants included in the primary analyses (1996–2009, during which social class was measured using the Registrar General's occupational classification), and for participants included in the sensitivity analysis (2001–2011, during which social class was measured using the National Statistics Socio-Economic Classification). In total, 155,311 participants were included in the primary analyses, with 6.7%, 31.7%, 14.5%, 24.9% 14.0% and 4.7% of participants classified as professional (I), managerial/technical (II), skilled non-manual (IIINM), skilled manual (IIIM), semi-skilled manual (IV), unskilled manual (V), and ‘other’, respectively. For social class, equivalised income, BMI, and alcohol consumption, there was approximately 0.2%, 26.2%, 12.4% and 0.9%, respectively, of participants with missing data. Similar distributions of socio-demographic characteristics were observed for the 155,311 participants included in the primary analyses (1996–2009), and the 115,622 participants included in the NS-SEC focused sensitivity analyses during years 2001–2011. Table 2 presents descriptive statistics for health outcomes of interest by year of survey. The total number of participants sampled in the survey years ranged from 4645 in 2009 to 16,443 in 1996. For the GHQ-12, between 8.6% and 15.9% of participants had scores ≥4, whilst between 24.7% and 58.7% had scores ≥1. For self-assessed health, between 18.3% and 30.1% of participants reported fair, bad or very bad health status each study year, whilst between 3.8% and 8.5% reported bad or very bad health. For EQ-5D utility scores, mean values ranged from 0.825 to 0.861 for the years where data were collected.

**Fig. 1:**
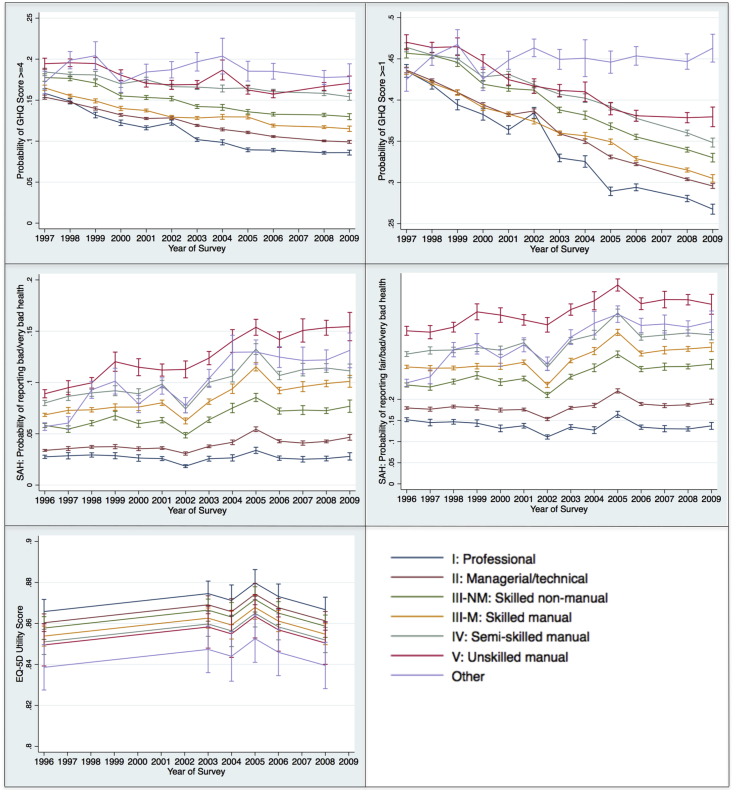
Changes in health outcomes by social class.

**Fig. 2:**
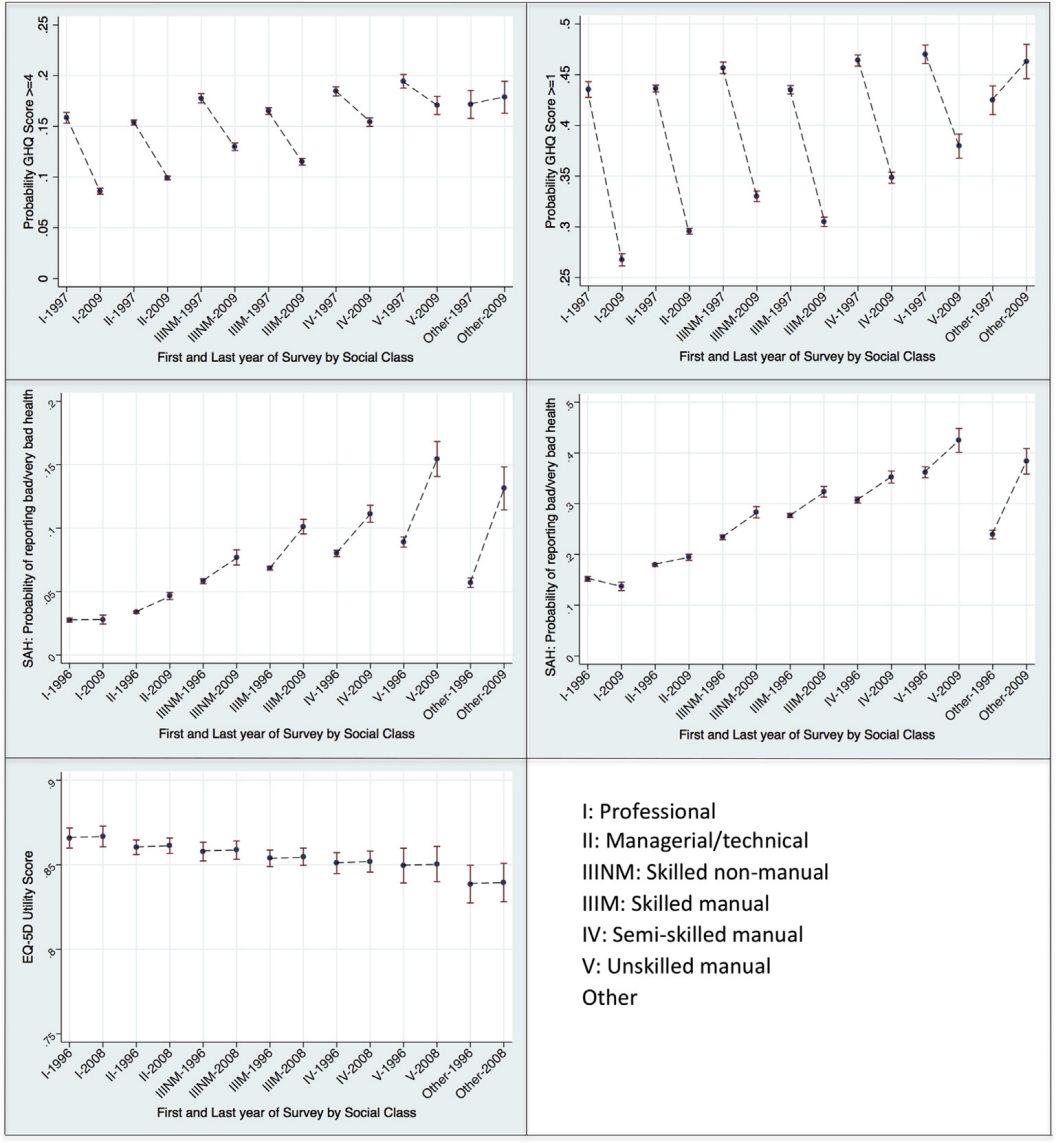
Time Trend Analysis by social class.

Table 3 shows the baseline results of the fully adjusted multi-level models. For the GHQ-12 and self-assessed health, the table shows the predicted probabilities of reporting each health outcome of interest by social class and year of survey, whilst for the EQ-5D, the table shows the predicted utility score by social class and year of survey. Fig. 1 provides a graphical representation of the health outcomes of interest for the survey years, with the trend showing how the health outcomes changed over the survey years. Fig. 2 compares the results of the first and final survey year by social class and shows whether over the time period of analysis values for each health outcome had significantly increased or decreased. Online files A and B show the results of the partially adjusted models 1 and 2, respectively.

For the GHQ-12, the probability of reporting scores ≥4 and ≥ 1 tended to be higher in those from lower social classes (measured using the Registrar General's occupational classification). The probability of reporting GHQ-12 scores ≥4 significantly reduced from 1997 to 2009 for all social classes. Figs. 1 and 2 highlight that there were also decreases in the probability of reporting a GHQ-12 score ≥1 from 1997 to 2009 for all social classes. For social class I, the predicted probability was 0.435 (95%CI: 0.428, 0.443) in 1997 and 0.268 (95%CI: 0.261, 0.274) in 2009; for social class II the predicted probability was 0.436 (95%CI: 0.433, 0.440) in 1997 and 0.296 (95%CI: 0.293, 0.299) in 2009; for social class III-NM the predicted probability was 0.457 (95%CI: 0.451, 0.462) in 1997 and 0.330 (95%CI: 0.325, 0.335) in 2009; for social class III-M the predicted probability was 0.435 (95%CI: 0.431, 0.439) in 1997 and 0.305 (95%CI: 0.300, 0.309) in 2009; for social class IV the predicted probability was 0.464 (95%CI: 0.458, 0.469) in 1997 and 0.348 (95%CI: 0.343, 0.354) in 2009; and for social class V the predicted probability was 0.470 (95%CI: 0.461, 0.479) in 1997 and 0.380 (95%CI: 0.368, 0.392) in 2009.

For SAH, the predicted probabilities of reporting bad or very bad health, and fair, bad or very bad health, were higher for those of lower social class. Fig. 1 shows the probability of reporting bad or very bad health remained relatively constant over the period 1996–2009 for those in social class I, but increased for those of lower social classes, with the greatest increase seen amongst those in social class V. For social class I, the predicted probability of reporting bad or very bad health was 0.028 (95%CI: 0.026, 0.029) in 1996 and 0.028 (95%CI: 0.024, 0.032) in 2009, whilst for social class V the predicted probability was 0.089 (95%CI: 0.085, 0.93) in 1996 and 0.155 (95%CI: 0.141, 0.168) in 2009. Figs. 1 and 2 show comparable trends for the predicted probabilities of reporting fair, bad or very bad health over the period 1996–2009.

EQ-5D utility scores remained comparable across survey years, and increased with higher socio-economic position (Table 3 and Fig. 1). Fig. 2 shows that from 1996 to 2008 there were non-significant changes in EQ-5D utility scores for each social class. For social class I, the predicted EQ-5D utility score was 0.866 (95%CI: 0.860, 0.872) in 1996 and 0.867 (95%CI: 0.861, 0.873) in 2009. Comparable values for the other social classes were 0.860 (95%CI: 0.856, 0.865) and 0.861 (95%CI: 0.857, 0.866) for social class II; 0.858 (95%CI: 0.852, 0.863) and 0.859 (95%CI: 0.853, 0.864) for social class III-NM; 0.854 (95%CI: 0.849, 0.959) and 0.855 (95%CI: 0.850, 0.860) for social class III-M; 0.851 (95%CI: 0.845, 0.857) and 0.852 (95%CI: 0.846, 0.858) for social class IV; and 0.850 (95%CI: 0.839, 0.860) and 0.850 (95%CI: 0.840, 0.861) for social class V.

The online files present the findings for the partially adjusted models and for the sensitivity analyses. The online files A and B demonstrate comparable findings to the baseline analyses in the partially adjusted models. The online files C and D show the changes in health outcomes by NS-SEC from 2001 onwards. Notably, less significant differences in health outcomes were observed between the NS-SEC groups than in the baseline analyses.

## 4 Discussion

Since the mid 1990's, there have been significant efforts in the UK, supported by government policies and funding, and a generally favourable economic climate, to tackle social inequalities in health. We examined trends in socio-economic inequalities in health against this policy and economic backdrop using responses to a range of self-reported measures of health status obtained from nationally representative samples over the period 1996–2009 (2001–2011 in sensitivity analysis). We found those in lower social classes were more likely to report poorer health than those in higher social classes, and that since the late 1990's these differences have persisted, with some evidence to suggest that they may have widened.

We examined responses to three different self-reported measures of health status. The GHQ-12 asks respondents to report the presence of symptoms associated with poor mental health (Goldberg et al., 1998). We applied two different thresholds in our analyses to allow examination of the impact of any symptoms (score ≥ 1) or multiple symptoms (score ≥ 4). The findings highlight the existence of a socio-economic gradient in the context of mental health. We found that since 1997 there have been general improvements in mental health across all socio-economic groups, although the greatest improvements were observed in the highest social classes. Previous studies have found a relatively weak and diminishing relationship between occupational measures of social class or income, and mental health (measured by the GHQ) (Fryers et al., 2003), with evidence suggesting unemployment has the strongest effect (Weich and Lewis, 1998; Wiggins et al., 2004). There is some evidence suggesting increases in income over time are strongly associated with positive mental well-being (Benzeval and Judge, 2001). During the period of our analyses, the majority of individuals in the UK experienced growth in their real income (Browne and Phillips, 2010), and this may explain the gradual and sustained improvement in GHQ scores across all social classes. Recent work in the UK suggests individuals in lower social classes are more likely to seek or receive treatment for mental health problems through health services (Jokela et al., 2013; Oliver et al., 2005). Despite this, we found no evidence of narrowing in socio-economic inequalities with regards to mental health, highlighting the need for understanding the underpinning causal relationships between social class and mental health within the context of a generally improved economic climate.

The GHQ-12 detects the presence of mental health symptoms (D. P. Goldberg et al., 1997), whilst SAH and the EQ-5D measure broader health status, encompassing physical, mental and social well-being (Dolan, 1997; Singh-Manoux et al., 2006). Over the time period of our analyses, we found that, with the exception of those in social class I, there was an increased likelihood of reporting bad or very bad health, alongside no significant changes in mean EQ-5D utility scores within social classes. Some published evidence suggests that SAH predominantly describes the experience of physical symptoms (Bailis et al., 2001), which may in part explain the differences seen across the measures. The differences between the SAH and GHQ-12 findings may be of interest, because suffering from physical illnesses increases the likelihood of suffering mental health problems (Das-Munshi et al., 2007), and consequently we might have expected to see an increased likelihood of reporting elevated GHQ-12 scores. Furthermore, SAH is predictive of future health outcomes (Idler and Benyamini, 1997; Miilunpalo et al., 1997), whilst the GHQ-12 provides a measure of current mental health problems (D. P. Goldberg et al., 1997). Consequently, there may be a time lag before changes in SAH translate into changes in mental health problems, which can only be assessed within the context of longitudinal studies. We used two different approaches to dichotomising SAH, and for both approaches found those in lower social classes more likely to report sub-optimal SAH, with some evidence for increased divergence between the social classes over time. In contrast, the EQ-5D analyses demonstrate the existence of a socio-economic gradient, but no significant changes in mean EQ-5D utility score within each social class from 1996 to 2008. Previous research has highlighted potential deficiencies with using the EQ-5D in health surveys of the general population, primarily driven by its limited sensitivity in detecting differences between mild health states and its ceiling effects (Macran et al., 2003). The majority of the UK population reports perfect health using the EQ-5D descriptive system, and consequently, there may be constraints in detecting differences between socio-economic groups and over time. Furthermore, the EQ-5D has been demonstrated to have poorer empirical validity than other utility measures in detecting differences in external indicators of health status (Petrou and Hockley, 2005).

In our multivariate analyses, we controlled for smoking, alcohol consumption and BMI. These lifestyle risk factors have higher prevalence amongst those of lower social class and previous work highlights the impact of these factors on HRQoL (Maheswaran et al., 2013). We undertook sensitivity analyses to examine changes in our health outcomes of interest by social class as classified by the NS-SEC. In general, the measures showed health outcomes improved from 2001 to 2011, with no clear evidence of widening socio-economic inequalities. The NS-SEC was not designed to classify individuals hierarchically (Chandola, 2000), which may explain why for some health outcomes, there were no significant differences across the social classes.

### 4.1 Strengths and limitations

The strength of the study lies in a combination of the large sample sizes used for analysis, use of data collected from nationally representative samples, examination of multiple self-reported health measures, examination of trends over extended periods, and the use of appropriate statistical methods to account for the hierarchical nature of the data and potential confounding. Whilst there are concerns regarding the use of self-reported measures of health, the GHQ-12 has been found to be a reliable tool for detecting psychiatric morbidity (Hardy et al., 1999), SAH is a powerful predictor of future health and use of health services (Idler and Benyamini, 1997; Miilunpalo et al., 1997), whilst the EQ-5D is the recommended tool for measuring the health impact of health interventions in England (NICE, 2004).

The study is not without its limitations. The HSE does not recruit individuals from institutionalised settings, many of whom will have poor health. Whilst we controlled for a range of determinants of socio-economic position, we examined changes in health outcomes by social class only. However, socio-economic position is a multi-dimensional concept influenced by a range of factors, and changes in burden of poor health over time may be comparable across other determinants of socio-economic position. Previous work has suggested that there may be systematic differences in responses to self-reported measures of health between individuals of different social groups, resulting in either over or under-estimation of health inequalities (Dowd and Todd, 2011). However, this would impact more on between-group differences than within-group differences, and therefore less likely to impact on our findings of secular trends, whilst the use of a range of self-reported health measures minimises potential biases by allowing greater measurement accuracy. Previous analysis have investigated health inequalities in relative terms, as a ratio of the burden of ill-health in one group relative to another, and/or in absolute terms, as the difference in the burden of ill-health between groups (King et al., 2012), with guidance highlighting that both should be presented (Kelly et al., 2007). However, the purpose of our analysis was to examine trends across all socio-economic groups, and therefore absolute and relative changes are not presented, but the data shown in Table 3 allows the reader to make comparisons between two groups in both absolute and relative terms. A further limitation is that single imputation of missing covariate values in the regression models may have underestimated the variance estimates of these parameters and may have inflated the statistical significance between the calendar years analysed. Nevertheless, the mean estimates are still unbiased and suitable for the trend analyses we aimed at.

## 5 Conclusions

Social inequalities in health are unjust, and tackling them remains a major policy objective. Over the last decade and a half, UK governments have systematically developed, implemented and resourced an extensive strategy to tackle social inequalities in health (Mackenbach, 2011). Despite these efforts, recent reviews have concluded these inequalities persist (DoH, 2009; Marmot et al., 2010). In our study, we assessed responses to a range of self-reported measures of health status from the general adult population in England, and found some evidence of increasing socio-economic inequalities in England over this period. Self-reported measures have the advantage of measuring current health, and policy changes may be expected to have a more immediate impact on these health outcomes, whilst investigating outcomes at the population level provides an essential approach to evaluating policy impacts (Fitzpatrick et al., 1992; Layard, 2010). We are unable to assess whether socio-economic related inequalities in self-reported health would have been even wider in the absence of government initiatives. However, recent evidence suggests many of the implemented strategies have had little or no impact on reducing social inequalities in health (Bauld et al., 2007; Judge and Bauld, 2006; Melhuish, Belsky, Leyland, Barnes, & National Evaluation of Sure Start Research (2008)). It is increasingly felt that income inequalities drive health inequalities (Marmot et al., 2010; Wilkinson and Pickett, 2006). During the time period of our analysis individuals generally experienced growth in their real incomes (Browne and Phillips, 2010); however, wage inequality worsened (Lindley and Machin, 2013), whilst income inequality, after tax and benefits, remained relative unchanged (Browne and Phillips, 2010). It is feasible that not enough was done in terms of reducing income inequality and this is what needs to be targeted at the policy level. Of concern is that our analysis does not fully capture all the consequences of the recent global financial crisis or the changing face of skilled labour driven by technological advances; both are likely to disproportionately impact those of lower socio-economic position. Our findings highlight the continued need to invest in improving health outcomes in the most disadvantaged members of society.

## Competing interests

All authors have completed the ICMJE uniform disclosure form at www.icmje.org/coi_disclosure.pdf and declare: no support from any organisation for the submitted work; no financial relationships with any organisations that might have an interest in the submitted work in the previous three years; no other relationships or activities that could appear to have influenced the submitted work.

## Funding

This research received no specific grant from any funding agency in the public, commercial or not-for-profit sectors.

## Figures and Tables

**Online File A:**
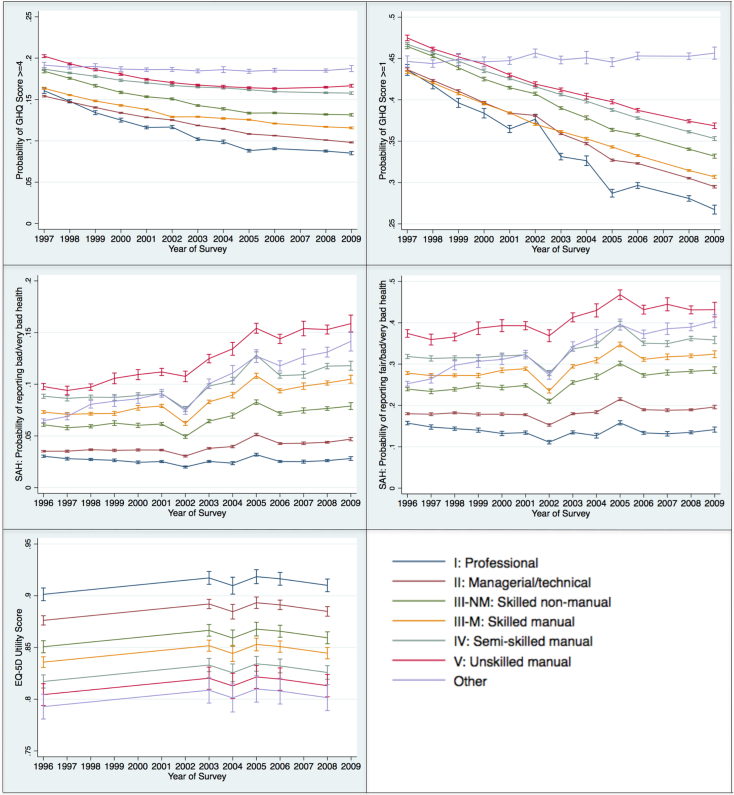
Results of multi-level analyses – Model 1.

**Online File B:**
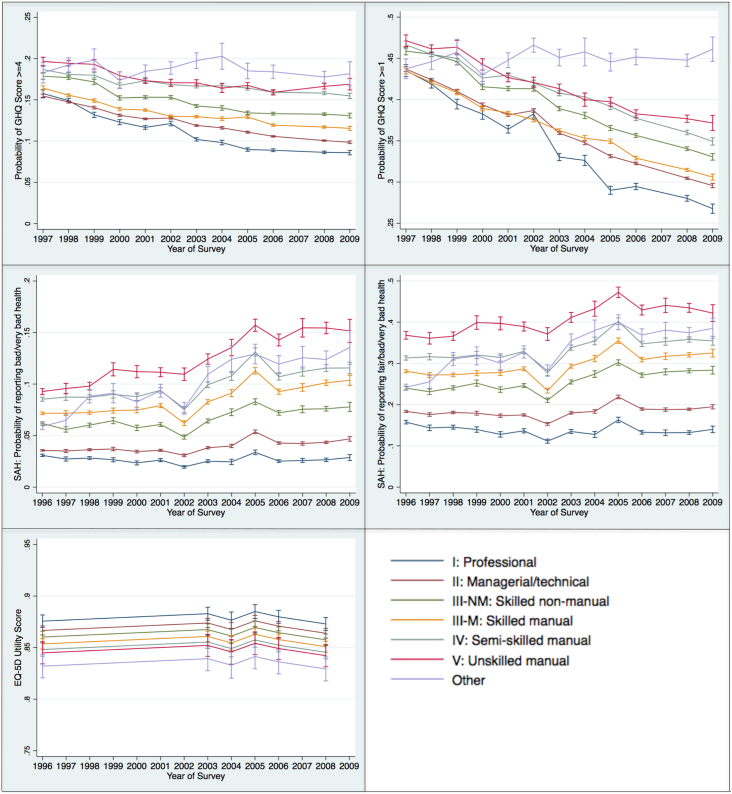
Results of multi-level analyses – Model 2.2.

**Online File C:**
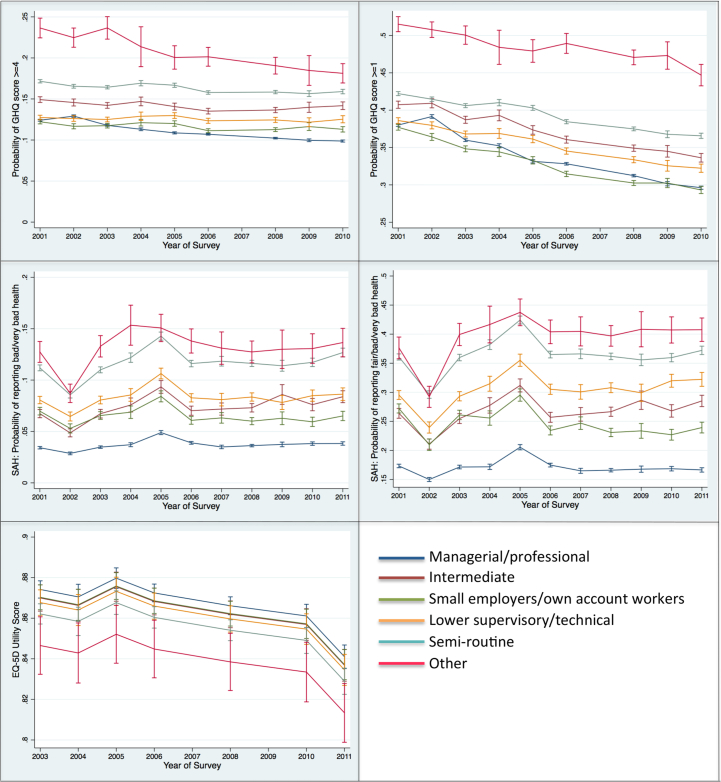
Changes in health outcomes by NS-SEC classification.3.

**Online File D:**
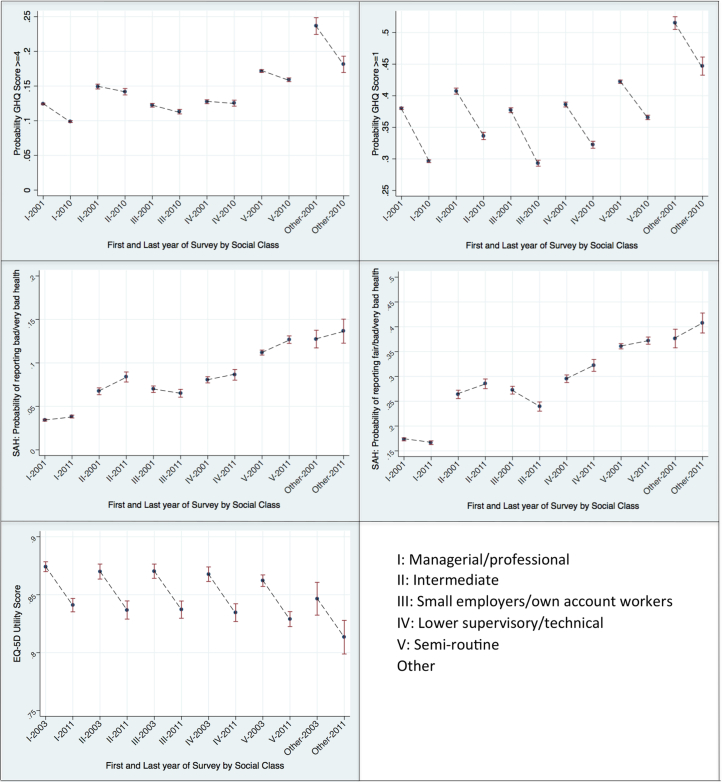
Time trend analysis by NS-SEC classification 4.

**Table 1:** Socio-demographic, economic and lifestyle characteristics of study population.

		Population for primary analyses (1996–2009)		Population for sensitivity analyses (2001–2011)	
		N	%	N	%
Total		155,311	100	115,622	100
Sex	Men	69,675	44.9	51,405	44.5
	Women	85,636	55.1	64,217	55.5
Age	16–24	19,752	12.7	14,692	12.7
	25–34	24,643	15.9	16,652	14.4
	35–44	28,290	18.2	20,722	17.9
	45–54	25,152	16.2	18,340	15.9
	55–64	22,369	14.4	17,651	15.3
	65–74	19,601	12.6	15,156	13.1
	75+	15,504	10.0	12,409	10.7
Ethnic group	White	143,185	92.2	105,359	91.1
	Black – Caribbean, Africa and Other	3143	2.0	2571	2.2
	Asian^a^	5930	3.8	5026	4.4
	Mixed	1180	0.8	1197	1.0
	Chinese and Other	1453	0.9	1086	0.9
	MISSING	420	0.3	383	0.3
Marital status	Single	31,903	20.5	23,328	20.2
	Married/Civil Partnerships	84,471	54.4	61,030	52.8
	Separated	3247	2.1	2521	2.2
	Divorced	9166	5.9	7011	6.1
	Widowed	13,395	8.6	9890	8.6
	Cohabitees	13,097	8.4	11,813	10.2
	MISSING	32	0.0	29	0.0
Highest Educational Qualification	NVQ4/NVQ5/Degree or equivalent	24,114	15.5	20,714	17.9
	Higher education below degree	16,577	10.7	12,352	10.7
	NVQ3/GCE A Level equivalent	18,662	12.0	15,291	13.2
	NVQ2/GCE O Level equivalent	35,546	22.9	26,137	22.6
	NVQ1/CSE other grade equivalent	8300	5.3	5946	5.1
	Foreign/other	5515	3.6	3144	2.7
	No qualification	46,055	29.7	31,576	27.3
	MISSING	542	0.4	462	0.4
Equivalised Income	Top Quintile	24,272	15.6	19,510	16.9
	4th Quintile	23,555	15.2	19,126	16.5
	3rd Quintile	23,516	15.1	19,001	16.4
	2nd Quintile	22,428	14.4	18,728	16.2
	Bottom Quintile	20,867	13.4	17,180	14.9
	MISSING	40,673	26.2	20,077	19.1
BMI	18.5 to <25	52,203	33.6	35,955	31.1
	<18.5	2209	1.4	1585	1.4
	25 to <30	51,871	33.4	37,354	32.3
	30 to <35	21,207	13.7	16,478	14.3
	35+	8609	5.5	7259	6.3
	MISSING	19,212	12.4	16,991	14.7
Cigarette Smoking	Never Ever Smoked	68,460	44.1	52,251	45.2
	Light <10/day	11,397	7.3	8256	7.1
	Moderate 10 to <20/day	15,397	9.9	10,628	9.2
	Heavy 20 + a day	11,433	7.4	7143	6.2
	Don't Know	234	0.2	192	0.2
	Ex-Smokers	47,187	30.4	36,037	31.2
	MISSING	1203	0.8	1115	1.0
Alcohol	Never Ever Drinker	10,131	6.5	8050	7.0
	Not at all in the last 12 months	1133	0.7	1074	0.9
	Once or twice a year	11,364	7.3	9087	7.9
	Once every couple of months	9921	6.4	7858	6.8
	Once or twice a month	18,958	12.2	14,372	12.4
	Once or twice a week	43,612	28.1	31,710	27.4
	Three or four days a week	23,071	14.8	16,682	14.4
	Five or six days a week	7219	4.7	5271	4.6
	Almost every day	21,730	14.0	15,292	13.2
	Ex-Drinker	6662	4.3	5318	4.6
	MISSING	1510	0.97	908	0.8
Social Class	I – Professional	10,466	6.7	–	–
	II – Managerial technical	49,275	31.7	–	–
	IIIN – Skilled non-manual	22,521	14.5	–	–
	IIIM – Skilled manual	38,730	24.9	–	–
	IV – Semi-skilled manual	21,807	14.0	–	–
	V – Unskilled manual	7260	4.7	–	–
	Others	4988	3.2	–	–
	MISSING	264	0.2	–	–
National Statistics Socio-Economic Classification (NS-SEC)	Managerial/professional	– – – – – – –	– – – – – – –	45,367	39.2
	Intermediate			10,203	8.8
	Small employers/own account workers			12,380	10.7
	Lower supervisory/technical			12,773	11.1
	Semi-routine			31,641	27.4
	Other			2957	2.6
	Missing			301	0.3

a Asian, includes Indian, Pakistani, Bangladeshi and Sri Lankan.

**Table 2:** Descriptive statistics of health outcomes of interest by study year.

			1996	1997	1998	1999	2000	2001	2002	2003
Total			16443	8582	15908	7798	7988	15647	10331	14836
GHQ Score (<4, ≥4)	Score ≥ 4	N (%)	–	835 (9.7)	2354 (14.8)	1274 (16.3)	1099 (13.8)	1967 (12.6)	1605 (15.5)	1840 (12.4)
	Score <4	N (%)	–	4486 (52.3)	12702 (79.8)	6167 (79.1)	6545 (81.9)	12683 (81.1)	8027 (77.7)	11974 (80.7)
	MISSING	N (%)	–	3261 (38.0)	852 (5.4)	357 (4.6)	344 (4.3)	997 (6.4)	699 (6.8)	1022 (6.9)
GHQ Score (<1, ≥1)	Score ≥ 1	N (%)	–	2318 (27.0)	6227 (39.1)	3525 (45.2)	2865 (35.9)	5620 (35.9)	4461 (43.2)	5095 (34.3)
	Score 0	N (%)	–	3003 (35.0)	8829 (55.5)	3916 (50.2)	4779 (59.8)	9030 (57.7)	5171 (50.1)	8719 (58.8)
	MISSING	N (%)	–	3261 (38.0)	852 (5.4)	357 (4.6)	344 (4.3)	997 (6.4)	699 (6.8)	1022 (6.9)
Self-Assessed Health	v.good, good, fair	N (%)	15516 (94.4)	8257 (96.2)	14839 (93.3)	7280 (93.4)	7473 (93.6)	14522 (92.8)	9738 (94.3)	13831 (93.2)
	bad, v.bad	N (%)	925 (5.6)	324 (3.8)	1068 (6.7)	517 (6.6)	511 (6.4)	1118 (7.1)	591 (5.7)	1002 (6.8)
	MISSING	N (%)	2 (<0.1)	1 (<0.1)	1 (<0.1)	1 (<0.1)	4 (0.1)	7 (<0.1)	2 (<0.1)	3 (<0.1)
Self-Assessed Health	v.good, good	N (%)	12502 (76.0)	7012 (87.7)	11757 (73.9)	5837 (74.9)	5908 (74.0)	11565 (73.9)	7972 (77.2)	11009 (74.2)
	fair, bad, v.bad	N (%)	3939 (24.0)	1569 (18.3)	4150 (26.1)	1960 (25.1)	2076 (26.0)	4075 (26.0)	2357 (22.8)	3824 (25.8)
	MISSING	N (%)	2 (<0.1)	1 (<0.1)	1 (<0.1)	1 (<0.1)	4 (0.1)	7 (<0.1)	2 (<0.1)	3 (<0.1)
EQ-5D	Available	N (%)	16047 (97.6)	–	–	–	–	–	–	13753 (92.7)
	MISSING	N (%)	396 (2.4)	–	–	–	–	–	–	1083 (7.3)
	EQ-5D utility Score	Mean (SD)	0.846 (0.002)	–	–	–	–	–	–	0.861 (0.002)

			2004	2005	2006	2007	2008	2009	2010	2011
Total			6704	10303	14142	6882	15102	4645	8420	8610
GHQ Score (<4, ≥4)	Score ≥ 4	N (%)	813 (12.1)	1160 (11.3)	1777 (12.6)	–	1296 (8.6)	740 (15.9)	1102 (13.1)	–
	Score <4	N (%)	5331 (79.5)	8089 (78.5)	11253 (79.6)	–	12925 (85.6)	3636 (78.3)	6370 (75.7)	–
	MISSING	N (%)	560 (8.4)	1054 (10.2)	1112 (7.9)	–	881 (5.8)	269 (5.8)	948 (11.3)	–
GHQ Score (<1, ≥1)	Score ≥ 1	N (%)	3932 (58.7)	3243 (31.5)	4718 (33.4)	–	3733 (24.7)	1944 (41.9)	2881 (34.2)	–
	Score 0	N (%)	2212 (33.0)	6006 (58.3)	8312 (58.8)	–	10488 (69.4)	2432 (52.4)	4591 (54.5)	–
	MISSING	N (%)	560 (8.4)	1054 (10.2)	1112 (7.9)	–	881 (5.8)	269 (5.8)	948 (11.3)	–
Self-Assessed Health	v.good, good, fair	N (%)	6203 (92.5)	9423 (91.5)	13114 (92.7)	6379 (92.7)	14007 (92.7)	4284 (92.2)	7760 (92.2)	7982 (92.7)
	bad, v.bad	N (%)	498 (7.4)	875 (8.5)	1025 (7.2)	500 (7.3)	1088 (7.2)	357 (7.7)	656 (7.8)	621 (7.2)
	MISSING	N (%)	3 (<0.1)	5 (<0.1)	3 (<0.1)	3 (<0.1)	7 (<0.1)	4 (0.1)	4 (<0.1)	7 (0.1)
Self-Assessed Health	v.good, good	N (%)	4929 (73.5)	7195 (69.8)	10464 (74.0)	5058 (73.5)	11180 (74.0)	3474 (74.8)	6226 (73.9)	6385 (74.2)
	fair, bad, v.bad	N (%)	1772 (26.4)	3103 (30.1)	3675 (26.0)	1821 (26.5)	3915 (25.9)	1167 (25.1)	2190 (26.0)	2218 (25.8)
	MISSING	N (%)	3 (<0.1)	5 (<0.1)	3 (<0.1)	3 (<0.1)	7 (<0.1)	4 (0.1)	4 (<0.1)	7 (0.1)
EQ-5D	Available	N (%)	6114 (91.2)	9211 (89.4)	12926 (91.4)	–	14117 (93.5)	–	7332 (87.1)	7517 (87.3)
	MISSING	N (%)	590 (8.8)	1092 (10.6)	1216 (8.6)	–	985 (6.5)	–	1088 (12.9)	1093 (12.7)
	EQ-5D utility Score	Mean (SD)	0.851 (0.003)	0.840 (0.002)	0.857 (0.002)	–	0.849 (0.003)	–	0.849 (0.003)	0.825 (0.003)

GHQ: General Health Questionnaire.

**Table 3:** Multi-level analyses of health outcomes of interest by social class (Registrar General's occupational classification).^a^

Health)outcome	Year	I:)Professional			II:)Managerial)technical			IIINM:)Skilled)non; manual			IIIM:)Skilled)manual			IV:)Semi; skilled)manual			V:)Unskilled)manual			Others		
		Mean	Lower) 95%)CI	Upper) 95%)CI	Mean	Lower) 95%)CI	Upper) 95%)CI	Mean	Lower) 95%)CI	Upper) 95%)CI	Mean	Lower) 95%)CI	Upper) 95%)CI	Mean	Lower) 95%)CI	Upper) 95%)CI	Mean	Lower) 95%)CI	Upper) 95%)CI	Mean	Lower) 95%)CI	Upper) 95%)CI
Probability)GHQ) Score)>=4	1997	0.158	0.153	0.164	0.154	0.151	0.156	0.178	0.173	0.182	0.165	0.162	0.168	0.184	0.180	0.189	0.194	0.188	0.201	0.172	0.158	0.185
	1998	0.149	0.145	0.152	0.147	0.146	0.149	0.177	0.174	0.179	0.155	0.154	0.157	0.181	0.178	0.184	0.196	0.191	0.200	0.199	0.188	0.209
	1999	0.132	0.128	0.135	0.140	0.138	0.142	0.171	0.167	0.175	0.149	0.147	0.152	0.181	0.176	0.185	0.195	0.187	0.202	0.204	0.187	0.221
	2000	0.122	0.119	0.126	0.132	0.130	0.134	0.155	0.152	0.159	0.140	0.137	0.143	0.170	0.166	0.174	0.181	0.174	0.187	0.171	0.159	0.183
	2001	0.116	0.114	0.119	0.128	0.126	0.129	0.153	0.151	0.156	0.137	0.136	0.139	0.175	0.172	0.178	0.170	0.166	0.175	0.184	0.175	0.194
	2002	0.123	0.119	0.126	0.128	0.127	0.130	0.152	0.149	0.155	0.129	0.127	0.131	0.166	0.163	0.170	0.169	0.163	0.174	0.187	0.177	0.197
	2003	0.102	0.100	0.104	0.119	0.118	0.120	0.142	0.140	0.145	0.128	0.126	0.130	0.166	0.163	0.169	0.169	0.164	0.174	0.197	0.186	0.208
	2004	0.099	0.095	0.102	0.114	0.113	0.116	0.141	0.138	0.145	0.130	0.127	0.133	0.164	0.160	0.168	0.187	0.175	0.199	0.204	0.182	0.226
	2005	0.089	0.087	0.092	0.111	0.109	0.112	0.136	0.133	0.139	0.129	0.127	0.132	0.165	0.161	0.168	0.162	0.157	0.168	0.185	0.173	0.197
	2006	0.089	0.087	0.091	0.106	0.105	0.107	0.133	0.131	0.135	0.119	0.117	0.121	0.160	0.157	0.163	0.157	0.153	0.162	0.185	0.176	0.195
	2008	0.086	0.084	0.088	0.100	0.099	0.101	0.132	0.130	0.134	0.117	0.115	0.119	0.158	0.156	0.161	0.167	0.162	0.172	0.178	0.169	0.186
	2009	0.086	0.083	0.089	0.099	0.097	0.101	0.130	0.126	0.134	0.115	0.112	0.118	0.154	0.150	0.158	0.171	0.162	0.179	0.179	0.163	0.194
Probability)GHQ) Score)>=1	1997	0.435	0.428	0.443	0.436	0.433	0.440	0.457	0.451	0.462	0.435	0.431	0.439	0.464	0.458	0.469	0.470	0.461	0.479	0.425	0.411	0.439
	1998	0.418	0.413	0.424	0.424	0.422	0.426	0.454	0.451	0.458	0.421	0.418	0.423	0.455	0.451	0.458	0.464	0.457	0.470	0.453	0.442	0.463
	1999	0.394	0.388	0.400	0.409	0.407	0.412	0.446	0.441	0.451	0.409	0.406	0.412	0.450	0.445	0.455	0.465	0.454	0.475	0.467	0.450	0.485
	2000	0.382	0.376	0.389	0.394	0.392	0.397	0.419	0.415	0.424	0.391	0.387	0.395	0.428	0.423	0.433	0.446	0.437	0.455	0.426	0.411	0.440
	2001	0.364	0.359	0.369	0.382	0.380	0.384	0.414	0.411	0.417	0.383	0.381	0.386	0.431	0.428	0.435	0.425	0.418	0.431	0.448	0.437	0.459
	2002	0.384	0.378	0.391	0.387	0.384	0.389	0.412	0.408	0.416	0.374	0.371	0.377	0.419	0.415	0.423	0.417	0.408	0.426	0.463	0.453	0.474
	2003	0.330	0.325	0.334	0.359	0.357	0.361	0.388	0.385	0.391	0.360	0.357	0.362	0.407	0.403	0.411	0.412	0.404	0.419	0.449	0.437	0.461
	2004	0.325	0.318	0.332	0.350	0.347	0.353	0.382	0.377	0.387	0.357	0.352	0.361	0.402	0.397	0.408	0.410	0.398	0.422	0.451	0.428	0.473
	2005	0.289	0.284	0.294	0.331	0.329	0.333	0.368	0.364	0.372	0.349	0.346	0.353	0.392	0.388	0.397	0.390	0.382	0.397	0.446	0.433	0.459
	2006	0.294	0.290	0.298	0.322	0.320	0.324	0.355	0.352	0.359	0.329	0.326	0.332	0.378	0.374	0.381	0.381	0.374	0.388	0.454	0.442	0.465
	2008	0.281	0.277	0.284	0.304	0.302	0.306	0.340	0.337	0.343	0.315	0.313	0.318	0.360	0.357	0.364	0.379	0.372	0.385	0.447	0.437	0.456
	2009	0.268	0.261	0.274	0.296	0.293	0.299	0.330	0.325	0.335	0.305	0.300	0.309	0.348	0.343	0.354	0.380	0.368	0.392	0.463	0.446	0.480
SAH:)Probability) of)reporting) bad/very)bad) health	1996	0.028	0.026	0.029	0.034	0.033	0.035	0.058	0.056	0.060	0.068	0.067	0.070	0.080	0.077	0.083	0.089	0.085	0.093	0.057	0.053	0.061
	1997	0.029	0.025	0.032	0.036	0.034	0.038	0.055	0.051	0.058	0.073	0.070	0.076	0.087	0.082	0.091	0.095	0.088	0.102	0.060	0.054	0.067
	1998	0.029	0.027	0.031	0.037	0.036	0.039	0.060	0.058	0.063	0.073	0.071	0.075	0.090	0.087	0.093	0.100	0.095	0.105	0.093	0.085	0.101
	1999	0.029	0.026	0.032	0.037	0.035	0.040	0.068	0.063	0.072	0.076	0.073	0.079	0.092	0.087	0.097	0.120	0.111	0.130	0.101	0.089	0.114
	2000	0.026	0.023	0.030	0.035	0.034	0.037	0.060	0.056	0.063	0.076	0.073	0.080	0.089	0.084	0.094	0.115	0.107	0.123	0.079	0.071	0.087
	2001	0.026	0.024	0.028	0.036	0.035	0.037	0.063	0.061	0.066	0.080	0.078	0.083	0.098	0.095	0.102	0.112	0.106	0.118	0.097	0.089	0.105
	2002	0.018	0.017	0.020	0.031	0.029	0.032	0.049	0.046	0.051	0.063	0.060	0.065	0.074	0.070	0.078	0.113	0.105	0.121	0.078	0.070	0.085
	2003	0.026	0.023	0.028	0.038	0.036	0.039	0.064	0.061	0.066	0.081	0.079	0.084	0.100	0.096	0.104	0.124	0.117	0.130	0.104	0.095	0.113
	2004	0.027	0.023	0.030	0.042	0.039	0.044	0.075	0.071	0.080	0.094	0.089	0.099	0.106	0.101	0.112	0.140	0.129	0.152	0.129	0.113	0.146
	2005	0.034	0.031	0.037	0.054	0.052	0.057	0.085	0.081	0.089	0.115	0.111	0.120	0.133	0.127	0.138	0.154	0.146	0.162	0.130	0.118	0.141
	2006	0.026	0.024	0.028	0.043	0.041	0.044	0.072	0.069	0.075	0.092	0.089	0.095	0.107	0.103	0.111	0.142	0.134	0.149	0.125	0.115	0.135
	2007	0.025	0.022	0.028	0.041	0.039	0.043	0.073	0.069	0.077	0.096	0.091	0.101	0.113	0.106	0.119	0.151	0.139	0.162	0.121	0.108	0.135
	2008	0.026	0.024	0.028	0.043	0.041	0.044	0.073	0.070	0.075	0.099	0.096	0.102	0.114	0.110	0.118	0.153	0.146	0.161	0.122	0.112	0.132
	2009	0.028	0.024	0.032	0.047	0.044	0.049	0.077	0.071	0.083	0.101	0.095	0.107	0.111	0.105	0.118	0.155	0.141	0.168	0.131	0.114	0.148
SAH:)Probability) of)reporting) fair/bad/very)bad) health	1996	0.152	0.147	0.156	0.179	0.177	0.182	0.234	0.229	0.239	0.277	0.273	0.281	0.307	0.301	0.313	0.362	0.351	0.373	0.239	0.230	0.248
	1997	0.145	0.137	0.153	0.177	0.172	0.181	0.229	0.222	0.237	0.274	0.267	0.280	0.315	0.307	0.324	0.359	0.343	0.375	0.253	0.237	0.269
	1998	0.147	0.141	0.152	0.183	0.179	0.186	0.242	0.236	0.248	0.274	0.269	0.279	0.317	0.310	0.323	0.371	0.359	0.382	0.317	0.300	0.334
	1999	0.144	0.136	0.151	0.180	0.175	0.185	0.257	0.248	0.265	0.278	0.272	0.285	0.322	0.313	0.332	0.407	0.388	0.426	0.331	0.307	0.355
	2000	0.131	0.123	0.139	0.175	0.170	0.179	0.241	0.233	0.248	0.278	0.271	0.285	0.316	0.306	0.326	0.399	0.382	0.416	0.298	0.278	0.318
	2001	0.137	0.132	0.143	0.176	0.172	0.179	0.249	0.244	0.255	0.288	0.282	0.293	0.334	0.327	0.341	0.388	0.375	0.400	0.329	0.312	0.345
	2002	0.111	0.106	0.116	0.153	0.150	0.157	0.210	0.204	0.217	0.234	0.228	0.240	0.276	0.268	0.284	0.376	0.359	0.393	0.284	0.269	0.300
	2003	0.134	0.129	0.140	0.180	0.176	0.183	0.253	0.248	0.259	0.292	0.286	0.297	0.339	0.332	0.346	0.412	0.399	0.426	0.347	0.330	0.365
	2004	0.127	0.119	0.135	0.186	0.181	0.191	0.275	0.265	0.284	0.314	0.306	0.323	0.353	0.342	0.363	0.433	0.411	0.454	0.380	0.352	0.408
	2005	0.164	0.157	0.172	0.220	0.215	0.225	0.306	0.299	0.314	0.358	0.351	0.366	0.404	0.396	0.413	0.470	0.456	0.485	0.401	0.382	0.421
	2006	0.134	0.129	0.139	0.189	0.185	0.192	0.271	0.265	0.278	0.308	0.302	0.314	0.347	0.340	0.354	0.426	0.412	0.440	0.375	0.358	0.392
	2007	0.130	0.123	0.138	0.185	0.180	0.190	0.277	0.269	0.286	0.316	0.307	0.325	0.352	0.342	0.362	0.436	0.416	0.455	0.378	0.357	0.399
	2008	0.130	0.125	0.135	0.187	0.184	0.190	0.278	0.272	0.283	0.319	0.314	0.325	0.357	0.350	0.364	0.435	0.423	0.448	0.371	0.356	0.386
	2009	0.137	0.129	0.145	0.194	0.188	0.200	0.283	0.272	0.294	0.323	0.313	0.334	0.353	0.341	0.364	0.425	0.401	0.448	0.384	0.358	0.409
EQ;5D)Utility) Score	1996	0.866	0.860	0.872	0.860	0.856	0.865	0.858	0.852	0.863	0.854	0.849	0.859	0.851	0.845	0.857	0.850	0.839	0.860	0.839	0.828	0.850
	2003	0.875	0.868	0.881	0.869	0.865	0.873	0.867	0.861	0.872	0.863	0.858	0.868	0.860	0.854	0.866	0.858	0.848	0.869	0.847	0.836	0.859
	2004	0.871	0.864	0.879	0.866	0.859	0.872	0.863	0.856	0.870	0.859	0.852	0.866	0.856	0.849	0.864	0.855	0.843	0.866	0.844	0.832	0.856
	2005	0.880	0.873	0.886	0.874	0.869	0.880	0.872	0.866	0.878	0.868	0.862	0.874	0.865	0.858	0.872	0.864	0.853	0.874	0.853	0.841	0.864
	2006	0.873	0.867	0.879	0.868	0.863	0.872	0.865	0.860	0.871	0.861	0.856	0.866	0.858	0.852	0.865	0.857	0.846	0.867	0.846	0.835	0.857
	2008	0.867	0.861	0.873	0.861	0.857	0.866	0.859	0.853	0.864	0.855	0.850	0.860	0.852	0.846	0.858	0.850	0.840	0.861	0.839	0.828	0.851

GHQ: General Health Questionnaire. SAH: Self-Assessed Health.

a Adjusted for age, sex, ethnicity, marital status, educational attainment, income (equivalised for household composition), smoking status, alcohol consumption and body mass index.
